# Supplementary material for: Interplay Between 3D Chromatin Architecture and Gene Regulation at the APOE Locus Contributes to Alzheimer’s Disease Risk
Source: Int J Mol Sci. 2025 Dec 27;27(1):302. doi: 10.3390/ijms27010302 (PMC12785688; doi:10.3390/ijms27010302)
Supplement: Supplementary file 1 [file ijms-27-00302-s001.zip › Supplimentary Materials.pdf]

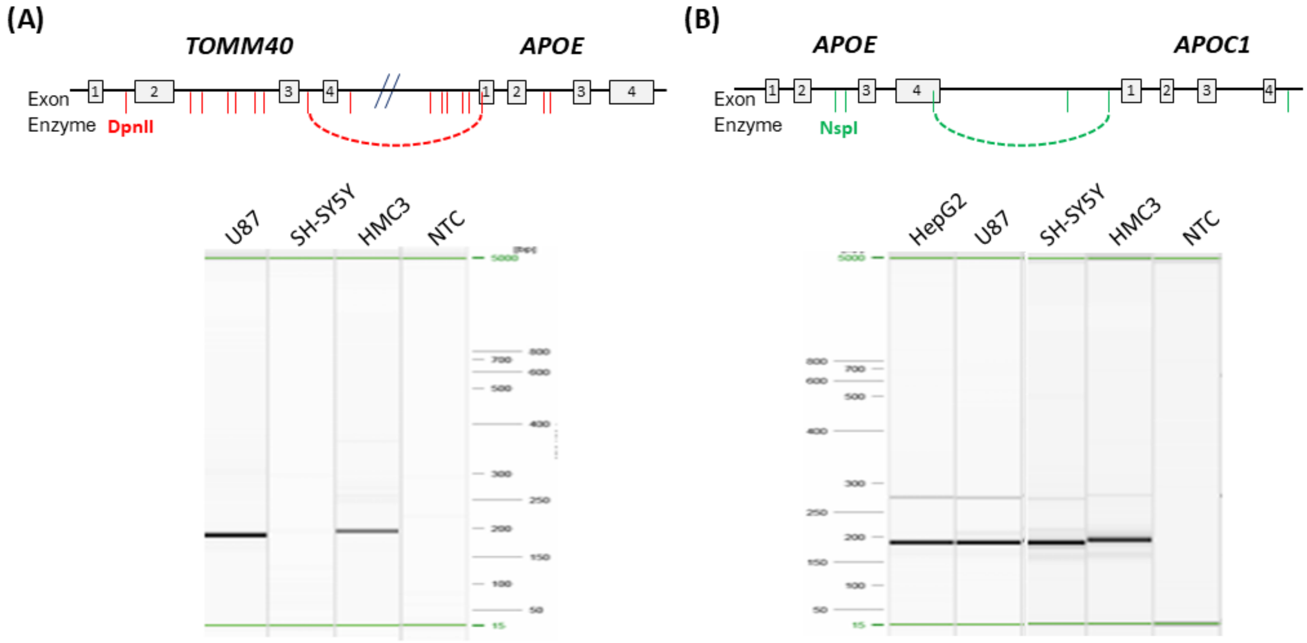

Figure S1. Chromatin interactions at the *APOE* locus were investigated using chromosome conformation capture (3C) in tissue-cultured human cell lines. The resulting ligation chimeric DNA products were detected and analyzed via PCR followed by digital gel electrophoresis. (A) Chromatin interactions spanning the *TOMM40*–*APOE* region were profiled in U87, SH-SY5Y, HMC3 cells, and non-template control (NTC). The interaction map depicts ligation of DpnII-digested fragments between *TOMM40* intron 3 (IVS3) and the *APOE* promoter. (B) Chromatin contacts between the *APOE* exon 4 CGI and *APOC1* promoter were examined in HepG2, U87, SH-SY5Y, HMC3 cells, and NTC. The interaction map illustrates ligation of NspI-digested fragments encompassing the *APOE* exon 4 CGI and *APOC1* promoter region.

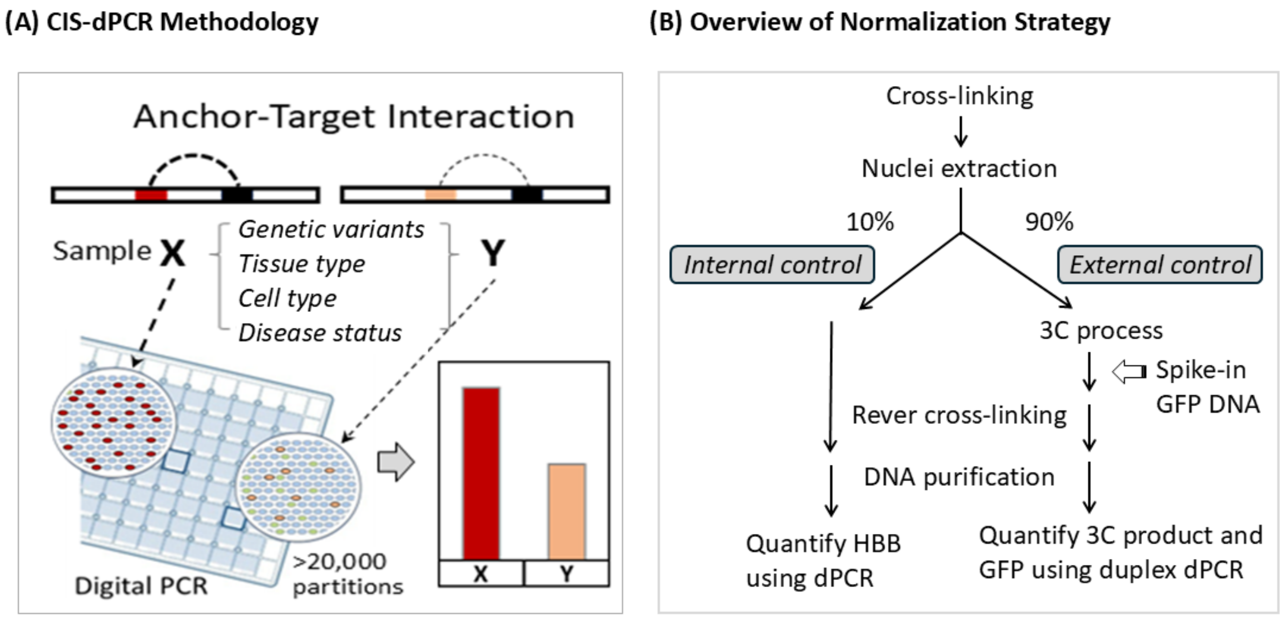

Figure S2. Schematic representation of the CIS-dPCR workflow for quantifying locus-specific chromatin interaction (CI) strength. (A) Schematic representation of the CIS-dPCR method for measuring CI strength across biological conditions. The thickness of dashed lines connecting anchor and target regions reflects relative interaction strength. (B) Experimental workflow outlining normalization strategy for accurate quantification of CI copy number. Internal normalization was performed using the Hemoglobin Subunit Beta (*HBB*) gene, which exhibits no copy number variation in the human genome. A fixed amount of exogenous Green Fluorescent Protein (*GFP*) DNA was spiked into each sample during the 3C process and quantified by duplex dPCR alongside the interaction-specific 3C products to provide external normalization.

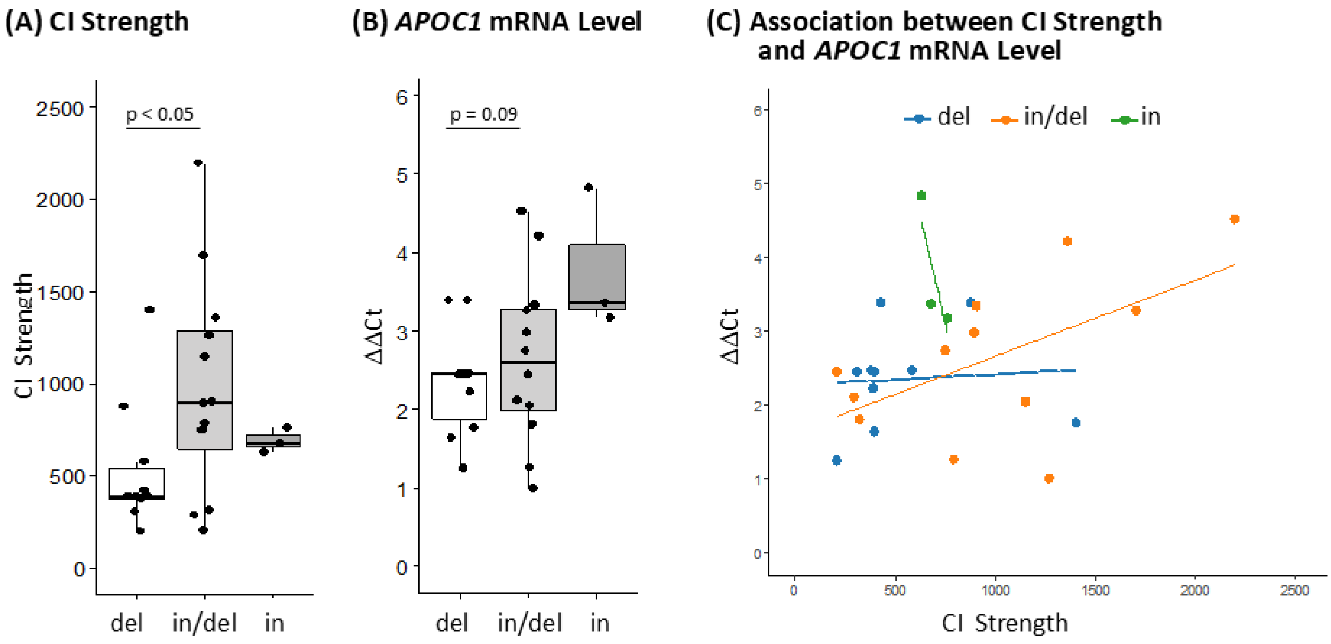

Figure S3. Impact of *APOC1* promoter insertion/deletion variant (rs11568822) genotype on chromatin interaction (CI) strength and *APOC1* expression in human PMB samples. (A) CI strength at the *APOE–APOC1* locus was stratified by *APOC1* rs11568822 genotype (del/del, n=10; in/del, n=12; in/in, n=3). CI strength differed significantly across groups, with in/del carriers showing the highest levels ($p < 0.05$). (B) *APOC1* mRNA levels (RT-qPCR, ΔΔCt method) were compared across the same genotypes. Expression showed genotype-dependent variation, though not statistically significant ($p > 0.09$). (C) Genotype-stratified correlations revealed a positive trend in in/del carriers (r = 0.56, p = 0.06), with no correlation observed in del/del or in/in groups. An independent samples t-test was used to compare across the biological variables.

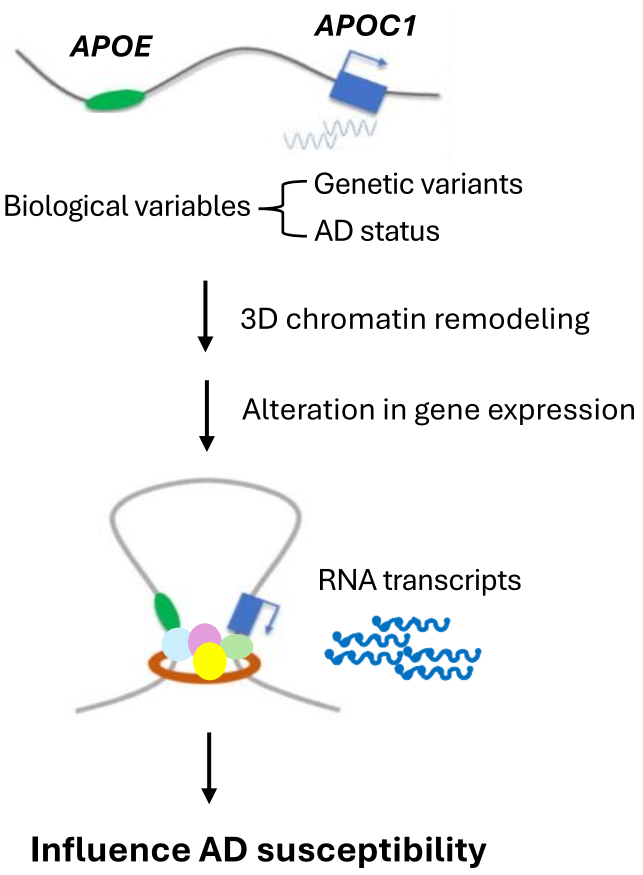

Figure S4. Proposed model of how *APOE* locus genes jointly influence AD risk. Schematic illustration of 3D chromatin remodeling at the *APOE* locus, influenced by genetic variants and AD status. This remodeling modulates promoter–enhancer interactions, alters gene expression profiles, and contributes AD risk.

Table S1. Raw chromatin interaction strength data obtained via 3C procedure and quantified via CIS-dPCR methodology

PMB ID	Disease	*APOE*	dPCR Target			Normalized 3C		
			HBB	***GFP***	**3C**	**3C**	**Mean**	**SD**
1	Ctrl	3/3	290	411	273	379	393	19
			290	408	290	407		
2			291	381	392	586	583	5
			291	391	398	579		
3			323	512	292	292	308	22
			323	524	331	324		
4			385	161	137	366	396	42
			385	221	220	426		
5			321	259	187	377	377	1
			321	264	191	378		
6	Ctrl	3/4	295	163	222	765	791	37
			295	139	203	817		
7			330	127	195	768	750	17
			330	123	183	747		
			330	133	194	735		
8			490	230	193	284	319	49
			490	174	182	354		
9			308	104	276	1419	1267	144
			308	127	282	1188		
			308	110	276	1350		
			308	116	285	1323		
			308	163	322	1058		
10			265	615	300	304	293	15
			265	772	349	282		
11	AD	3/3	279	351	251	423	426	4
			279	389	281	430		
12			294	87	127	822	876	36
			294	104	164	892		
			294	104	166	895		
			294	101	160	894		
13			379	276	245	387	391	7
			379	248	226	396		
14			350	556	288	224	205	26
			350	742	322	187		
15			226	90	157	1274	1405	116
			226	97	199	1497		
			226	106	210	1444		
16	AD	3/4	311	80	346	2286	2198	286
			311	97	341	1878		
			311	74	339	2430		
17			316	79	256	1686	1358	302
			316	98	214	1140		
			316	66	193	1541		
			316	114	233	1065		
18			263	140	280	1262	1152	176
			263	112	221	1244		
			263	154	232	949		
19			403	57	273	1961	1702	267
			403	61	255	1718		
			403	75	260	1428		
20			449	139	344	914	903	104
			449	147	316	794		
			449	110	299	1001		
21	AD	4/4	361	946	431	209	207	3
			361	1871	836	205		
22			326	195	262	681	679	17
			326	197	269	693		
			326	184	249	688		
			326	220	283	654		
23			231	196	147	542	631	126
			231	147	146	720		
24			319	130	192	763	762	10
			319	120	179	775		
			319	137	199	752		
			319	133	195	758		
25			288	135	200	847	896	63
			288	124	189	873		
			288	109	184	967		

Table S2. Primers, probes, and TaqMan assays

Method	Target	Primer	Sequence 5'-	Size (bp)	Comment
**Hot-Start PCR**					
	TOMM40–APOE interaction	T40_F	CCCTGTCTAACTAGGCTGTACT	424	Forward primer for preamplification
		T40_R	GGGGGTGGTCAAAAGACCTCTA		Reverse primer for preamplification
		T40_NF	GAGATGAGAGTTGGTGTGGGGTTGGAGTGG	186	Forward primer for nested PCR
		T40_NR	GGGAGCCCTATAATTGGACAAGTC		Reverse primer for nested PCR
	APOE–APOC1 interaction	APOC1_F	GCAGGCCCAGCAGATAC	270	Forward primer for preamplification
		APOC1_R	CCAATTTCTGCCTCCAAAGAAAG		Reverse primer for preamplification
		APOC1_NF	AAGACATGCACCGGTAATTAGAT	190	Forward primer for nested PCR
		APOC1_NR	GAAATGAAATCCACAAGCAGACA		Reverse primer for nested PCR
**Digital PCR**					
	APOE–APOC1 interaction	3C_F	GTGGAAGACATGCACCGGTAATTAGAT	133	Forward
		3C_R	GCCTGGTGGTTAAAGATTGA		Reverse
		3C_probe	/56-FAM/AGATAACCT/ZEN/ AACGGATTAGGTCAGGG/3IABkFQ/		Internal probe
	HBB	HBB_F	CAACGTGCTGGTCTGTGT	86	Forward
		HBB_R	CAGCCACCACTTTCTGATAGG		Reverse
		HBB probe	/5Cy5/CTGGCCCAT/TAO/ CACTTTGGCAAAGAA/3IAbRQSp/		Internal probe
	GFP	GFP_F	GATGCGGGTGTTGGTGTAG	95	Forward
		GFP_R	GCTACGGCTTCTACCACTTCG		Reverse
		GFP probe	/5Cy5/CGTT+GTTGA+T+GGCGTGCA+ GGAAG/3IAbRQSp/		Internal probe
**TaqMan Assay**					
	APOC1 RNA		ThermoFisher (Hs00155790_m1)		
	ACTB RNA		ThermoFisher (Hs01060665_g1)		
